# Characterizing Relationship of Microbial Diversity and Metabolite in Sichuan *Xiaoqu*

**DOI:** 10.3389/fmicb.2019.00696

**Published:** 2019-04-12

**Authors:** Qiuxiang Tang, Guiqiang He, Jun Huang, Chongde Wu, Yao Jin, Rongqing Zhou

**Affiliations:** ^1^College of Light Industry, Fermentation Engineering, Sichuan University, Chengdu, China; ^2^Key Laboratory for Leather Chemistry and Engineering of the Education Ministry, Sichuan University, Chengdu, China; ^3^National Engineering Research Centre of Solid-state Brewing, Luzhou, China

**Keywords:** *Xiaoqu*, microbial diversity, MiSeq high-throughput sequencing, fermentation characteristics, volatiles, correlation

## Abstract

*Xiaoqu* is a fermentation starter used in the production of *Xiaoqu jiu*, which is also a traditional Chinese liquor. The quality and microbial community characteristics of *Xiaoqu* is closely related with the yield and flavor feature of fresh *Xiaoqu jiu*. The present study aims to explore the mystery behind microbial diversity and volatiles of *Xiaoqu* through polyphasic detection methods such as the Illumina MiSeq platform and the metabolite analyzing method. Results showed that differences in microbial community diversity among samples were significant. The hydrolytic ability was positively correlated with α- and β-diversity of bacteria, but negatively correlated with that of fungi. *Staphylococcus* and *Weissella* were the dominant bacteria, while *Rhizopus* and *Candida* were the dominant fungi. The abundance of bacteria in sample No3 ranged from 33.66 to 91.53%, while sample No4 the abundance of fungi ranged from 58.51 to 48.72%. The difference of microbial community diversity resulted in a discrepancy of volatile profiles and interaction relationship among the genus. Twenty-four dominant bacteria and seven dominant fungi were correlated with 20 different volatiles. This study provides a scientific perspective of the uniformity and stability of *Xiaoqu jiu* and might aid in controlling its manufacturing process.

## Introduction

As a conventional solid fermentation of *Baijiu*, *Xiaoqu jiu* is a very popular Chinese liquor in Asia. *Xiaoqu jiu* can be classified into three main types: *Qing-xiang*; *Yu ping shao*; and *Guilin-sanhua jiu*. Of these, *Qing-xiang* (light in flavor) *Xiaoqu jiu* is different from *Daqu jiu* such as *Jiang-*, *Nong-* and *Qing-xiang* *Baijiu*, although the main materials are the same, which includes grain sorghum (*Sorghum bicolor* L. Moench) and rice hulls (Su et al., 2010). The technology is characterized as follows: a short fermentation period, provides a higher yield, while less *Xiaoqu* is used during the starter process, in contrast to the production technology used for *Daqu jiu* (Gou et al., 2015). The *Xiaoqu* involved in various types, relies on raw materials and production technology, where rice and rice bran are the main raw materials. Sichuan *Xiaoqu*, a typical *Xiaoqu*, often adds *Muqu*, matured *Xiaoqu* produced during the last year. Some Chinese herbs also have a long history of being added to this process (Zheng and Han, 2016). The production of *Xiaoqu* and *Xiaoqu jiu* are both carried out in an open environment, while pure *Aspergillu*s culture is inoculated into bran when the process starts for *Fuqu* (Zheng and Han, 2016). Therefore, the microbial community diversity depends on the process parameter and region niches featured, endowing a unique fragrance to the *Xiaoqu jiu*. The microbial community structure of *Xiaoqu* originates from the three different regions and is characterized by culture-dependent and culture-independent technology (Wu et al., 2017). The bacterial community structure is more complex than fungus’, and the abundance of dominant bacteria varies within samples, but is similar to that of dominant fungus’. The bacterial community structure is also affected by the fermentation pattern (Li et al., 2016; Xiao et al., 2017). For example, the bacterial community diversity in traditional starters is similar, but different to that produced by the automatic starter-making disk machine. The dominant genera are *Weissella*, *Acetobacter* and *Gluconobacter* in the former, while *Acetobacter*, *Acinetobacter*, and *Klebsiella* are dominant in the latter, while both *Lactobacillus* and *Pediococcus* are the dominant genera in all starters (Wang et al., 2018). In general, the difference of microbial community diversity and flavor among *Xiaoqu* may lead to a striking divergence of the end product. The starter is therefore regarded as the soul of *Baijiu* (Zheng and Han, 2016; Jin et al., 2017). It is therefore necessary to develop a foundation for new *Xiaoqu* techniques, such as automatic or intelligent culture techniques, that understands the relationship between dominant microbes with volatile components and physicochemical properties.

In the present study, sequencing of four typical *Xiaoqu* was performed using the Illumina MiSeq platform. The volatiles profile was determined using HS-SPME-GCMS. To the best of our knowledge, the correlation of microbial and volatile profiles and the interaction of the dominant genera with *Xiaoqu* are also revealed for the first time.

## Materials and Methods

### Sample Collection and Preparation

Samples were supplied by four typical *Xiaoqu* enterprises located in Sichuan, Chongqing, Yunnan, and Hubei, respectively, and labeled as No1, No2, No3, and No4. Sampling was carried out according to the process described by Zhang et al. (2014), and was then transferred into sterile bags and stored at −20°C until analysis.

### Physiochemical Properties Determination

The physicochemical property of *Xiaoqu* samples was determined according to the general methods of analysis for *Daqu* (Ministry of Light Industry of China, 2011). These properties includes moisture, fermenting power, esterifying power, saccharifying power, and liquefying power.

### DNA Purification and PCR Amplification

Total genomic DNA was extracted directly from these samples using the E.Z.N.A.®Soil DNA Kit (OMEGA). DNA extraction and purification were carried out using the method previously described (Wang H.-Y. et al., 2008). Using a NanoDrop 2000 spectrophotometer (Thermo Fisher Scientific, United States) the concentrations and quality of purified DNA was determined, and then PCR amplified. The V3–V4 hypervariable regions of the 16S rRNA genes were amplified by universal primers (F: 5′- ACTCCTACGGGAGGCAGCA-3′, R: 5′- GGACTACHVGGGTWTCTAAT-3′), PCR was carried out in 25 μL using the following program: preliminary denaturation for 5 min at 98°C, followed by 24 cycles of 98°C for 30 s, 52°C for 30 s (annealing), and 72°C for 1 min (extension). A final annealing step of 72°C for 5 min was performed. The ITS region of the fungal 18S r RNA gene was targeted using ITS1 universal primers (F: 5′- GGAAGTAAAAGTCGTAACAAGG-3′, R: 5′- GCTGCGTTCTTCATCGATGC-3′). The PCR program for amplification of the ITS region was the same, except the number of cycles was 28.

The resulting PCR products were purified using the QIA quick Gel Extraction kit Qiagen (Germany) and only PCR products without primer dimers and contaminant bands were used for the following high-throughput sequencing. After gel purification, the PCR product was quantified by NanodropNC2000 spectrophotometer (Thermo Fisher Scientific, Shanghai, China). Next the PCR products of all samples were mixed into a single pool with equal molecular weight based on the quantification results and the mixture of amplicon were sequenced on an Illumina MiSeq 2 × 300 platform (Illumina Inc., San Diego, CA, United States) and 200∼450 base pair (bp) paired – end reads were generated.

### High-Throughput Sequencing and Sequence Analysis

All sequencing data have been deposited at the Sequence Read Archive of the National Center for Biotechnology Information, with SRA accession number: **PRJNA491669**. BioSample accessions of sequences: **SAMN10081877 (No1)**, **SAMN10081878 (No2)**, **SAMN10081879 (No3)**, **SAMN10081880 (No4)**. Pairs of reads were overlapped based on a method described previously (Magoč and Salzberg, 2011). Sequencing reads were assigned to each sample according to the individual unique barcode. Sequences were analyzed with the FLASH (Fast Length Adjustment of Short reads, version 1.2.11) and UPARSE pipeline (Caporaso et al., 2010). The reads were first filtered by FLASH quality filters. Then, the UPARSE pipeline was used to detect operational taxonomic units (OTU) at 97% similarity (Blaxter et al., 2005; Edgar, 2010). For each OTU, a representative sequence was selected and used to assign taxonomic composition using the RDP classifier (Wang et al., 2007). Estimated species richness was determined with a rarefaction analysis. Rarefaction curves and the estimates of Chao1, Ace, and Simpson/Shannon, representing the species abundance, the amount of unique OTUs found in each sample, and the microbial diversity, respectively, were generated by the Mothur software (Schloss et al., 2011). Bray–Curtis distances were calculated by QIIME pipeline and the similarity and difference between the communities were depicted by Hierarchical cluster analysis and Venn diagram.

### Analyzing of Volatiles

Volatiles analyzing was carried out using a Trace GC Ultra gas chromatograph-DSQ II mass spectrometer (Thermo Electron Corp., Waltham, United States) equipped with an HP-INNOWAX capillary column (30.0 m × 0.25 mm × 0.25 μm, Agilent Technology, CA, United States) and a flame ionization detector (FID). Mass spectrum was generated in the electron impact (EI) mode at 70 eV ionization energy using the full scan mode (40 to 500 amu).

The GC operation condition was as follows: an inlet temperature of 250°C, split ratio of 10:1, Helium (purity: 99.999%) carrier gas flow of 1 mL/min. The oven temperature was maintained at 40°C for 5 min, followed by an increase of 5°C/min to 220°C, and held for 5 min. The constituents were tentatively identified by matching the mass spectrum with the NIST05 spectrum database and verified by comparison of their Kováts retention indices (RI) with the RI reported in literatures, calculated using C_8_–C_30_ n-alkanes.

### Bioinformatics and Statistical Analysis

Sequence data analyses were mainly performed using QIIME (v1.8.0) and R packages (v3.2.0). OTU-level alpha diversity indices, such as Chao1 richness estimator and Shannon diversity index, were calculated using the OTU table in QIIME. The communities were depicted by Rarefaction curves, Venn. Hierarchical cluster analysis and Heatmaps were plotted via R packages. The taxonomy compositions and abundances were visualized using MEGAN (Huson et al., 2011). Beta diversity analysis was performed to investigate the structural variation of microbial communities across samples using non-metric multidimensional scaling (NMDS) (Ramette, 2007). The physiochemical properties were mainly performed using Origin (v8.5). Linear discriminant analysis effect size (LEfSe) was executed in the Galaxy website^1^ to analyze the difference in metabolite content among samples (Ter Braak and Smilauer, 2002; Jiang et al., 2015). Redundancy analysis (RDA) was executed in CANOCO 4.5 for Windows (Biometris, Wageningen, Netherlands) to determine the chemical properties with significant influences on microbial communities (Ter Braak and Smilauer, 2002; Jiang et al., 2015). Figures were generated with CanoDraw 4.0 (Biometrics Wageningen, Netherlands). Co-occurrence analysis was performed by calculating Spearman’s rank correlations between predominant taxa. Correlations with | RHO| > 0.6 and *P* < 0.01 were visualized as a co-occurrence network using Cytoscape (v3.2.1) (Shannon et al., 2003). Microbial functions were predicted by PICRUSt (Phylogenetic Investigation of Communities by Reconstruction of Unobserved States) (Langille et al., 2013), based on the KEGG PATHWAY Database^2^ and the network of microbiology (Shannon et al., 2003; Ramette, 2007; Huson et al., 2011).

## Results

### Difference of Microbial Community Diversity Among *Xiaoqu* Samples

The numbers of effective sequences of the four samples ranged from 37066 to 765799, and from 57511 to 78224 for bacteria and fungi, respectively. The average number and ratio of high-quality sequences was 40556 and 84.62%, as well as 64884 and 96.93% for bacteria and fungi, respectively (Supplementary Table S1). The rarefaction curves based on the OTU number (Supplementary Figure S1), approaching the saturation plateau, suggested that the sequencing depth was adequate to represent the microbial structure of samples (Supplementary Figure S1).

Analysis of α-diversity and β-diversity were conducted after normalization. The indexes of sample α-diversity, including the abundance and diversity index, were quite scattered and comparable among samples, and no general rule was actually revealed (Supplementary Table S2). The difference of α-diversity was surely related to the complex factors. Both Chao1 and ACE in sample No1 were 1064.04, and Shannon and Simpson were 6.89 and 0.96, respectively. It suggested that abundance and diversity were the highest for bacterial communities of No1 (Shannon, 1948; Simpson, 1949; Chao, 1984; Chao and Yang, 1993). The abundance of fungi in No3 was the highest, and Chao1 and ACE were 110.25 and 113.54, respectively. The number and proportion of unique bacterial OTUs was the highest by Venn diagram (Supplementary Figure S2) but there was no significant difference in the total number or unique number of the fungal OTUs among the four *Xiaoqu* samples.

### Microbial Community Diversity Profiles

**FIGURE 1:**
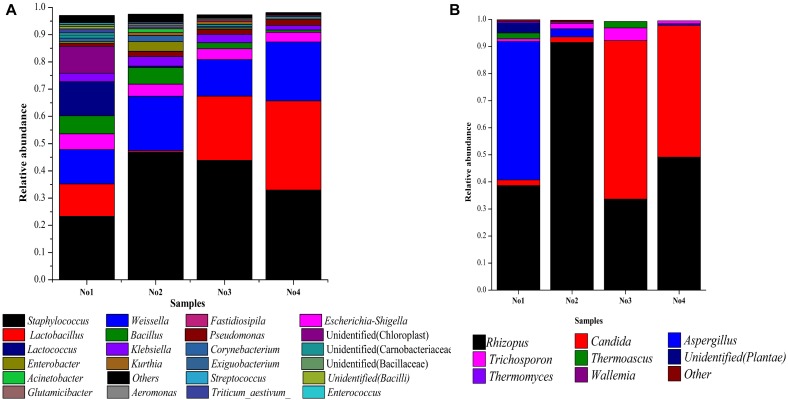
**(A)** Microbial community structure in each sample (bacteria). **(B)** Microbial community structure in each sample (fungi). Stacked bar graphs illustrate the abundance of genera.

The dissimilar microbial communities among these samples are shown in Figure 1. The abundance of *Staphylococcus* and *Weissella* were dominant in all samples. The abundance of *Staphylococcus* and *Weissella* were 23.32, 46.77, 43.84 and 32.96%, as well as 12.58, 19.98, 13.39, and 21.63% in No1, No2, No3, and No4, respectively. The abundance of *Lactobacillu*s was also dominant, in No1, No3, and No4 and were 11.89, 23.61, and 32.75%, respectively, but, in No2 it was only 0.61%. In addition, *Lactococcus* and an unidentified genus belonging to *Carnobacteriaceae* in No1 were both predominant. Among eight genera of the fungi identified, *Rhizopus* and *Candida* were dominant. The abundance of *Rhizopus* accounted for 38.63, 91.52, 33.66, and 49.13% in No1, No2, No3, and No4, respectively, and that of *Candida* in No3 and No4, accounted for 58.51 and 48.72%.

**FIGURE 2:**
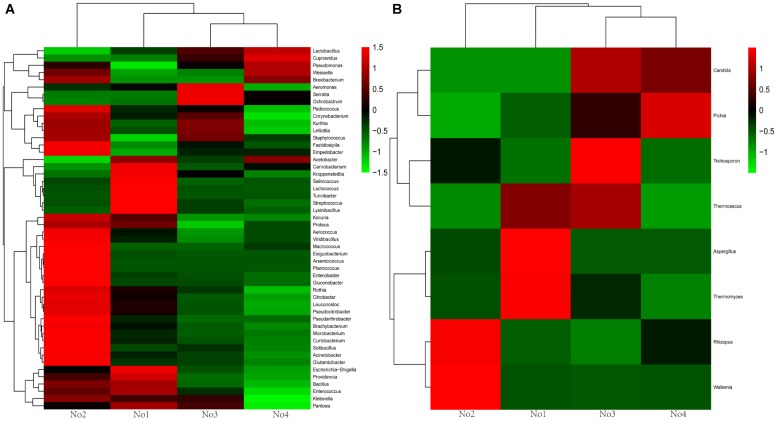
Heat map of genera level community composition in combination with cluster analysis. **(A)** Bacteria heat map. **(B)** Fungi heat map.

A hierarchically clustered heatmap analysis based on the microbial community profiles is shown in Figure 2. For bacteria and fungi, sample No2 was classified an alone cluster, and the remaining three samples were divided into another cluster, in which sample No1 was grouped as a subcluster and sample No3 and No4 as another subcluster. There were two dominant genera including *Wallemia* and *Rhizopus* in sample No2. *Aspergillus* and *Thermomyces* were dominant in sample No1.

**FIGURE 3:**
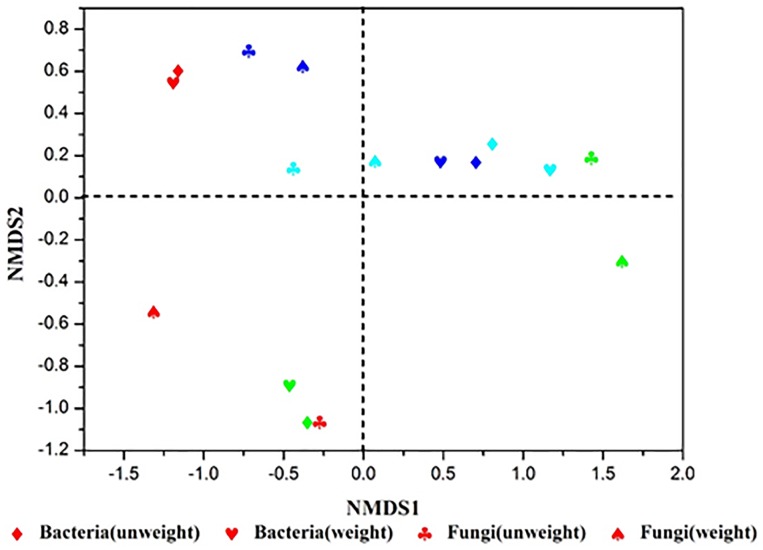
Results of NMDS sequencing of Xiaoqu. No1, No2, No3, No4 are represented by red, green, sky blue, dark blue, respectively. The stress value of NMDS for microbial are all 0.0000.

Additionally, NMDS and hierarchical clustering analyses, which examine relationships between microbial communities, were used to determine whether those OTUs identified, differentiated among different types of starters (Figure 3). The comparisons by NMDS (genus level similarity) using the Bray–Curtis similarity metric, indicated that the identified taxa significantly partitioned the starter samples into three distinct groups, with sample No3 clustering with sample No4, while sample No1 and No2 were separated by non-metric multi-dimensional scaling, which was consistent with the results obtained from the heatmap and hierarchical clustering analyses.

### Fermentation Characteristics and Metabolic Characteristics of *Xiaoqu*

Generally, physicochemical properties are also important indicators used to evaluate the quality of *Xiaoqu* (Zhang et al., 2012). Table 1 shows the differences of properties among these samples. The esterifying power was the best in No4, reaching 123.56 mg/50g ⋅ 7d, which was significantly higher than the other samples, while No1 was 1.95 mg/50g ⋅ 7d, the lowest. Sample No1 and No4 had excellent saccharifying power and were 1771.50 mg/g • h and 1071.38 mg/g • h, respectively. The order of liquefying power was No4(1.07 g/g • h) >No1(1.05 g/g • h) >No2(0.56 g/g • h) >No3(0.30 g/g • h). A slight difference of fermentability among samples was observed, in which that of No4 was the lowest, and samples ranged from 7.90 g/0.5 g • 72 h to 7.43 g/0.5 g • 72 h.

**Table 1:** Differences in physicochemical properties of four *Xiaoqu* samples.

Sample	Moisture(%)	fermenting power(g/0.5 g ⋅ 72 h)	esterifying power (mg/50 g ⋅ 7 d)	saccharifying power (mg/g ⋅ h)	liquefying power (g/g ⋅ h)
No1	4.67 ± 0.00	7.90 ± 0.22	1.95 ± 0.08	1771.50 ± 0.00	1.05 ± 0.08
No2	6.18 ± 0.00	7.50 ± 0.35	54.01 ± 6.98	964.50 ± 22.63	0.56 ± 0.03
No3	6.33 ± 0.00	7.80 ± 0.79	25.12 ± 12.67	274.88 ± 17.50	0.30 ± 0.02
No4	7.61 ± 0.00	7.43 ± 0.55	123.56 ± 35.13	1071.38 ± 121.45	1.07 ± 0.10

**FIGURE 4:**
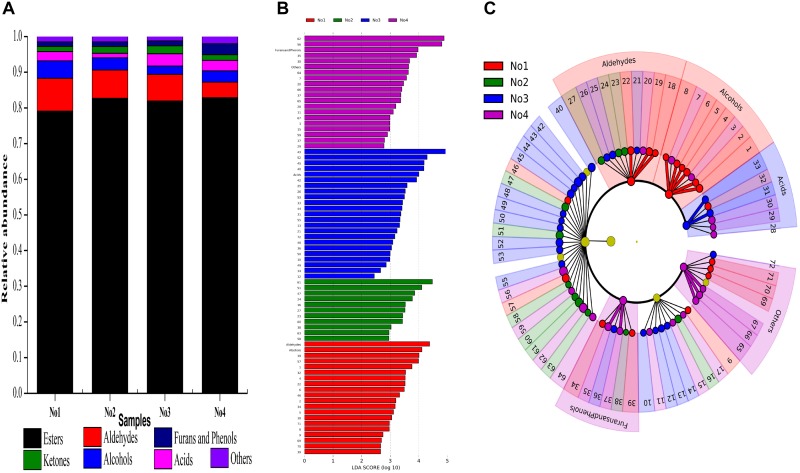
**(A)** Proportions of volatile substances in the sample. Bar graph **(B)**, and Plot cladogram **(C)** results on the abundance of volatiles. Volatiles differentiality are represented by a linear discriminant analysis, coupled with effect size (LEfSe) (LDA > 2, *P* < 0.05). No1, No2, No3, No4 are represented by red, green, blue, purple, respectively. 1: hexanol 2: 2-heptenal 3: 3-methyl-1-pentanol 4: 1-octen-3-ol 5: 2,3-butanediol 6: benzenemethanol 7: benzeneethanol 8: *trans-*nerolidol 9: 3-octen-2-one 10: 3-nonen-2-one 11: 3,5-octadien-2-one 12: 6-dodecanone 13: 6,10-dimethyl-2-undecanone 14: *cis-*geranylacetone 15: 1-(1H-pyrrol-2-yl)-ethanone 16: 6,10,14-trimethyl-2-pentadecanone 17: dodecalactone 18: 5-ethyl-1-cyclopentene-1-carbaldehyde 19: 2-octen-1-al 20: 3-furaldehyde 21: decanal 22: benzaldehyde 23: 2-nonenal 24: 2-decenal 25: 2-butyl-2-octenal 26: 2,4-nonadienal 27: 2-undecenal 28: acetic acid 29: pentanoic acid 30: hexanoic acid 31: octanoic acid 32: sorbic acid 33: nonanoic acid 34: 2-pentylfuran 35: 2-methoxyphenol 36: phenol 37: 2-methoxy-4-vinylphenol 38: dibenzofuran 39: 2,3-dihydro-benzofuran 40: methyl hexanoate 41: methyl 4-hexanoate 42: methyl heptanoate 43: methyl nonanoate 44: octyl formate 45: methyl decanoate 46: methyl benzate 47: methyl dodecanoate 48: methyl tridecanoate 49: methyl 8-oxooctanoate 50: methyl isomyristate 51: methyl tetradecanoate 52: methyl Z-11-tetradecenoate 53: nonanoic acid, 9-oxo-, methyl ester 54: methyl pentadecanoate 55: nonanedioic acid, dimethyl ester 56: methyl hexadecanoate 57: methyl palmitoleate 58: 7,10-hexadecadienoic acid, methyl ester 59: methyl heptadecanoate 60: methyl octadecanoate 61: elaidic acid, methyl ester 62: linoleic acid, methyl ester 63: 6,9,12-octadecatrienoic acid, methyl ester 64: linolenic acid, methyl ester 65: tetradecane 66: caryophyllene 67: heptadecane 68: naphthalene 69: 2-methylnaphthalene 70: 1,7-dimethylnaphthalene 71: 2,2,5,5-tetramethyl-1,1-biphenyl 72: 1-methoxy-4-(1-propenyl)-benzene.

The quality and flavor feature of *Xiaoqu* starter was closely related to their fresh *Xiaoqu jiu’s*, especially for flavor characteristics. A total of 72 volatiles were detected in these samples, and according to their chemical structure characteristics, they were divided into seven categories, including esters (25), acids (6), alcohols (8), ketones (9), aldehydes (10), furans and phenols (6), as well as others (8). The relative abundance of various components in different samples is shown in Figure 4A. Esters accounted for a similar proportion in the four samples, and a large proportion of volatile substances, all of which are around 80%.

To explore the variation of the volatile’s composition among these samples, we performed LEfSe tests to detect differences in abundance of volatile taxa (including Alcohols, Ketones, Aldehydes, Acids, Furans and Phenols, Esters, and Others) across samples. Sixty-eight types of volatile substances were significantly different in these samples (LDA > 2, *P* < 0.05) (Figure 4B). The contents of six alcohols and three aldehydes in sample No1 were significantly higher than the other samples which included hexanol, 2-heptenal, 1-octen-3-ol, 2,3-butanediol, benzenemethanol, *trans-*nerolidol,5-ethyl-1-cyclopentene-1-carbaldehyde, 2-octen-1-al, and benzaldehyde, respectively. The content of octanoic acid and nonanoic acid was significantly higher in sample No3. Additionally, the contents of 2-methoxyphenol and 2-methoxy-4-vinylphenol in sample No4 were significantly higher compared with the other samples, while that of tetradecane, caryophyllene and heptadecane were significantly higher (LDA > 2, *P* < 0.05) (Figure 4C).

### Correlation Analysis of Metabolites and Microflora and Network Relationships Between Microbes Themselves

**FIGURE 5:**
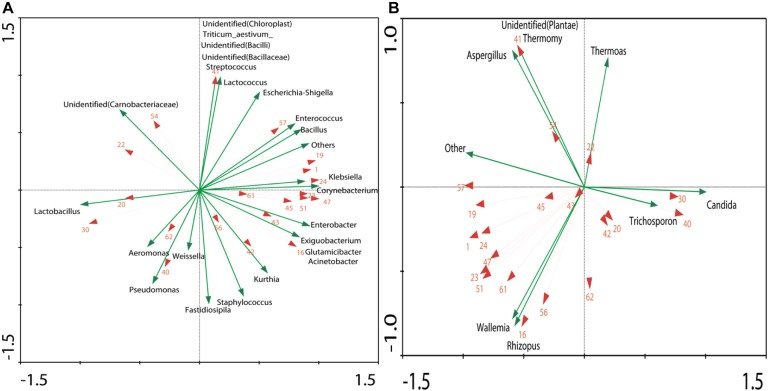
RDA of dominant genera (OTU > 1%) and dominant volatiles (The volatiles were consistent with Figure 4): **(A)** Correlation between volatiles and bacteria genera. **(B)** Correlation between volatiles and fungi genera.

The result obtained by RDA showed that 20 different volatile components were correlated with 24 dominant genera of bacteria and seven dominant genera of fungi (Figure 5).The length of the arrow indicates the extent to which the community structure, with taxa distribution, can be explained by the environmental variables. The angles between the arrows indicate the degree to which the environmental variables are correlated. The analysis was based on the correlation of microbial members that were used to estimate the relationship between microbes, which is important because of its possible utilization for the design of synetic microbial communities or bioaugmentation strategies (Faust et al., 2015; Kumar et al., 2016).

**FIGURE 6:**
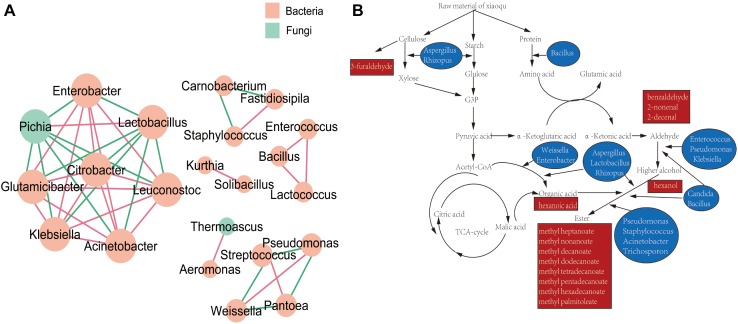
**(A)** Association network diagram of dominant genera. **(B)** Metabolic map of dominant genera in *Xiaoqu*.

The resulting network (Figure 6A) was composed by six cycles of fungi and bacteria, consisting of 22 nodes (genera), and 43 edges (the relationship between genera). Genera of *Enterobacter*, *Lactobacillus*, *Leuconostoc*, *Acinetobacter*, *Klebsiella*, *Glutamicibacter*, *Pichia*, and *Citrobacter* composed the biggest cycle. According to metabolic map of dominant genera in *Xiaoqu* (Figure 6B), *Rhizopus*, *Aspergillus*, and *Bacillus* worked on the beginning of fermentation, which degraded cellulose, starch and protein into substances available for subsequent fermentation. *Bacillus*, *Enterococcus*, *Candida*, *Pseudomonas*, and *Klebsiella* promoted the production of alcohols (Donaldson et al., 2010). *Aspergillus*, *Lactobacillus*, *Rhizopus*, *Weissella*, and *Enterobacter* could produce organic acids (Urdaneta et al., 1995; Sabatini et al., 2008; Takebe et al., 2016). Genera of *Staphylococcus*, *Lactobacillus*, *Weissella*, *Bacillus*, *Pseudomonas*, *Acinetobacter*, *Rhizopus*, *Candida*, *Aspergillus*, and *Trichosporon* have been reported to have a synthetic esters function (Sharma et al., 2001; Hasan et al., 2006).

## Discussion

The present study is the first report that compares the different *Xiaoqu*, exploring the mystery behind the microbial community. Dominant bacteria and fungi composition in these samples were consistent with previous studies (Gou et al., 2015; Wu et al., 2017). Bacteria included *Staphylococcus*, *Lactobacillus*, *Weissella*, *Escherichia-Shigella*, *Bacillus*, *Lactococcus*, *Klebsiella*, *Unidentified (Chloroplast)*, *Pseudomonas*, *Enterobacter*, *Corynebacterium*, *Unidentified* (*Carnobacteriaceae*), while fungi included *Rhizopus*, *Candida*, *Aspergillus*, *Trichosporon*. Dominant microbes were the same, in which lactic acid bacteria was the predominant bacteria (Jin et al., 2017), *Candida* and *Trichosporon* were the dominant yeast, while mold included *Rhizopus*, *Trichoderma*, *Aspergillus*.

Nevertheless, great differences in community diversity among these samples are shown in Figure 1–3, even though they were widely applied in the *Xiaoqu jiu* brewing process in China. The bacterial and fungal community composition in both No3 and No4 were very similar, but visible differences between sample No1 and sample No2 were observed. Various factors such as the process parameter, microhabitat contained in niches, Wang et al. (2018) may have contributed to these differences. Furthermore, the characterization of the dominant microbial constitution was obtained by the result of NMDS. For example, the abundance of *Rhizopus* and *Candida* in both sample No3 and sample No4 were similar and the distance of both in NMDS (shown in Figure 3) was relatively short. But it is worth noting that the quality of *Xiaoqu* and its unique features do not only lie in the abundance of various dominant fungi. For instance, the abundance of yeast and mold in sample No3 and No2 was higher than the other samples, respectively, but the hydrolyzing ability of starch and ester should be strongest, according to the speculation of the result obtained by the similar single strain (Guo et al., 1999; Vahvaselkä and Laakso, 2014). In fact, the quality and unique features of *Xiaoqu* is dependant on the coordination of the dominant species’ structure, as well as the interaction of functional species (Zheng et al., 2011; Zhu and Tramper, 2013; Zhu et al., 2015). As shown in Table 1, the relationship between saccharifying- and liquefying- power among these samples was positive, and the community structure of both No3 and No4 was similar (Figure 3) so that their fermentation characteristics were similar (Table 1). The result suggested that the fermentation characteristic can be regulated by the microflora.

Usually, the microbial community diversity of *Xiaoqu* contributed to the volatiles abundance and diversity. For example, the microbial community diversity in sample No1 was the largest so that the significant difference of volatile abundance between sample No1 and other samples was observed (Figure 1, 4C). Figure 4A shows that the proportion of volatiles in No3 and No4 were very similar to the community structure. As shown in Figure 4, the content and constituent of volatiles between sample No1 and sample No2 was different since the community structure was very dissimilar (Figure 1–3). Therefore, the present results provide scientific information on the regulation of the *Xiaoqu* manufacturing process as well as an improvement on the quality and flavor of *Xiaoqu jiu*.

In order to better understand the relationship between microorganism and volatile components, the formation of volatiles in *Xiaoqu* was analyzed visually with a metabolic map (Figure 6B). Different from *Daqu*, the abundance of *Rhizopus* in *Xiaoqu* was especially important, since it is closely related to starch hydrolysis, alcohol fermentation and flavor. In addition, *Saccharomyces* was mainly responsible for the alcohol yield, except for the flavor feature of *Xiaoqu jiu* (Wang C.-L. et al., 2008; Wu et al., 2009; Xiu et al., 2012; Li et al., 2014). It has been reported that *Bacillus* in starter can produce specific flavor compounds such as 2,3-butanediol, 3-hydroxy-2-butanone, 2-methylpropionic acid, and 3-methylbutanoic acid (Wu and Xu, 2012; Zhang et al., 2013; Meng et al., 2015). Traditionally, *Rhizopus* and *Aspergillus* have been widely involved in many fermented foods, and are also considered as important functional microbes. The abundance of *Lactobacillus* in both No3 and No4 was much higher than that in sample No1 and sample No2, which can produce either alcohol or lactic acid by hetero-fermenting (Zaunmüller et al., 2006). Except for *lactobacillus*, *Weissella*, *Streptococcus*, *Lactococcus*, *Enterococcus*, et al., *Xiaoqu* had a higher abundance (Figure 1, 6A). Lactic acids, one of the major metabolites of these lactic acid bacteria (Jin et al., 2017), can not only inhibit some bacteria, but is also an ethyl lactate precursor. Therefore, these bacteria were important functional microbes. As shown in Figure 5, major acids were positively correlated with *Lactobacillus*, *Weissella*, and *Candida*, while the content of major esters was positively related to *Staphylococcus*, *Weissella*, *Bacillus*, *Rhizopus*, and *Wallemia*. Methyl hexadecanoate was positively correlated to *Staphylococcus*, *Weissella*, *Pseudomonas*, and *Rhizopus*, but negatively related to *Candida* and *Trichosporon*. Methyl tetradecanoate and elaidic acid, methyl ester was positively related to *Bacillus*, *Klebsiella*, *Enterobacter*, *Corynebacterium*, *Acinetobacter*, *Enterococcus*, and *Rhizopus* (Figure 5B, 6). Therefore, the abundance of acids in sample No2 should be lower than that in other samples, consistent with the result detected. It was similar for sample No3 and No4, but the content of volatiles, as well as esters, especially that of methyl hexadecanoate were different. Figure 6B, clearly shows that *Staphylococcus, Bacillus*, and *Rhizopus* were beneficial and promoted the synthesis of esters. Among these samples, the content of alcohols, ketones, aldehydes, and esters in No2 were 67.53, 36.85, 156.86, and 1632.99 μg/kg, respectively, and were higher than that in other samples. This might be because of more bacterial species and a higher abundance of *Rhizopus* (91.5%) contained in sample No2 (Figure 2). The content of volatiles was the highest in sample No2 which contained the most esters species and the least acids species as the abundance of *Lactobacillus* was lower, while the abundance of *Staphylococcus* and *Rhizopus* was highest (Figure 1). *Staphylococcus* was the highest in No2, accounting for 46.77% of bacterial microorganisms, where Staphylococcus converted hexanol into esters such as ethyl valerate and hexyl acetate (Karra-Châabouni et al., 2006). However, the hexanol content in No2 was the highest among the four samples. That may have been caused by the high levels of 6,10,14-trimethyl-2-pentadecanone, which was reported to be bacteriostatic for *Staphylococcus* (Morteza-Semnani et al., 2012). 2-Nonenal was degraded by *Lactobacillus* in the fermentation process (Vermeulen et al., 2007), which may explain why 2-nonenal content in No2 was high as it had a low content of *Lactobacillus*. *Lactobacillus* was just 0.61% in No2, but was 11.89, 23.61, and 32.96% in No1, No3, and No4, respectively. *Klebsiella* has been reported to promote synthesis of alcohols (Donaldson et al., 2010). Meanwhile, hexanol and 2-octen-1-al both had a positive correlation with the genera of *Klebsiella*, as revealed by the RDA analysis (Figure 5A). Coincidentally, hexanol and 2-octen-1-ol were significantly different in No1 *Xiaoqu* volatiles (LDA > 2, *P* < 0.05) (Figure 4C).

## Conclusion

The difference in microbial community structures among different *Xiaoqu* samples was significant. The saccharifying- and liquefying-power were positively correlated with α- and β-diversity of bacteria but was also positively correlated with that of fungi. *Staphylococcus* and *Weissella* were dominant bacteria, while *Rhizopus* and *Candida* were dominant fungi, and abundance ranged from 33.66 to 91.53% for the former, while it was 58.51 and 48.72%, respectively in sample No3 and sample No4, for the latter. The difference of microbial community diversity resulted in the discrepancy of the volatile profile and the interaction relationship among thegenus. Twenty-four dominant bacteria and seven dominant fungi were correlated with 20 different volatiles. The aim was to lay a theoretical foundation in order to better understand the contribution of dominant flora and metabolic components, to the fermentation process and production style of *Xiaoqu*.

## Author Contributions

QT performed the experiments, analyzed the data, and prepared the manuscript. GH contributed to the manuscript discussion. JH guided the experiments. CW and YJ contributed to the manuscript revision. RZ contributed to the experimental design, manuscript revision, and overall support of this study.

## Conflict of Interest Statement

The authors declare that the research was conducted in the absence of any commercial or financial relationships that could be construed as a potential conflict of interest.

## Footnotes

1 http://huttenhower.sph.harvard.edu/galaxy/root

2 http://www.genome.jp/kegg/pathway.html
