# Supplementary material for: Characterizing Relationship of Microbial Diversity and Metabolite in Sichuan Xiaoqu
Source: Front Microbiol. 2019 Apr 12;10:696. doi: 10.3389/fmicb.2019.00696 (PMC6473189; doi:10.3389/fmicb.2019.00696)
Supplement: Supplementary file 1 [file Table_1.DOCX]

Supplementary Material

# Supplementary Figures and Tables

## Supplementary Table

**Supplementary Table 1 |** OTUs distribution on difference level of different samples

| Samples | No. of sequences | | | | Proportions (%) | |
| --- | --- | --- | --- | --- | --- | --- |
|  | Effective sequences | | High quality sequences | |  |  |
|  | Bacteria | Fungus | Bacteria | Fungus | Bacteria | Fungus |
| No1 | 37644 | 59370 | 29022 | 58497 | 77.10 | 98.53 |
| No2 | 39148 | 57511 | 35052 | 54038 | 89.54 | 93.96 |
| No3 | 37066 | 72009 | 31357 | 69904 | 84.60 | 97.08 |
| No4 | 76579 | 78224 | 66793 | 77098 | 87.22 | 97.16 |

## Supplementary Figure


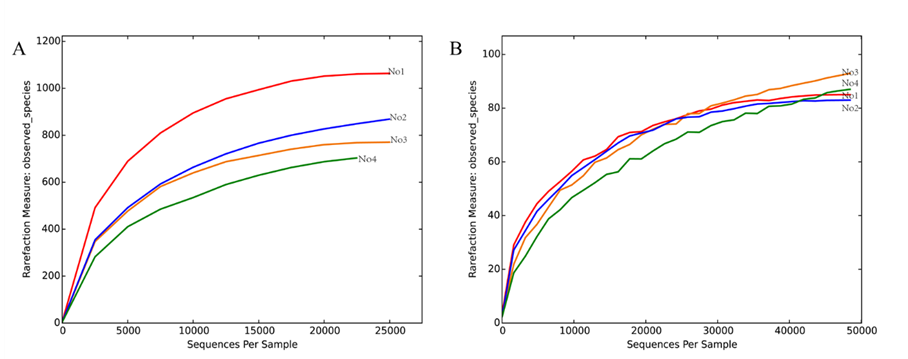


**Supplementary Figure 1.** Bacteria **(A)** and fungi **(B)** rarefaction analysis of the different samples. Rarefaction curves of OTUs clustered at 97% sequence identity for different samples.

## Supplementary Table

**Supplementary Table 2 |** difference of α-diversity among samples

| No. | Abundance index | | | |  | Diversity index | | | |
| --- | --- | --- | --- | --- | --- | --- | --- | --- | --- |
|  | Chao 1 | | ACE | |  | Shannon | | Simpson | |
|  | Bacteria | Fungus | Bacteria | Fungus |  | Bacteria | Fungus | Bacteria | Fungus |
| No1 | 1064.00 | 85.00 | 1064.00 | 85.00 |  | 6.89 | 1.97 | 0.96 | 0.62 |
| No2 | 925.38 | 83.00 | 975.00 | 83.00 |  | 5.20 | 1.54 | 0.83 | 0.50 |
| No3 | 771.00 | 110.25 | 771.36 | 113.54 |  | 5.17 | 1.61 | 0.86 | 0.57 |
| No4 | 820.44 | 102.00 | 849.21 | 108.09 |  | 4.69 | 1.36 | 0.86 | 0.54 |

## Supplementary Figure


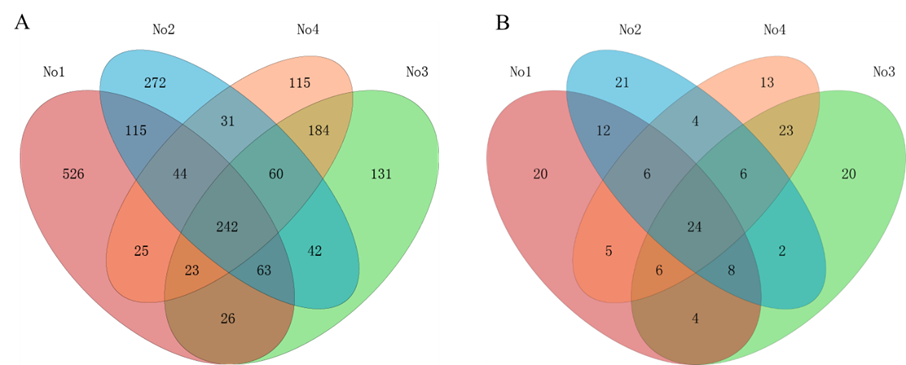


**Supplementary Figure 2.** Venn diagram showing the unique and shared OTUs in different starters. **(A)** Bacteria OTUs. **(B)** Fungi OTUs
